# Crystal structure of *trans*-1,4-bis­[(tri­methyl­sil­yl)­oxy]cyclo­hexa-2,5-diene-1,4-dicarbo­nitrile

**DOI:** 10.1107/S1600536814014251

**Published:** 2014-07-19

**Authors:** Florian Glöcklhofer, Johannes Fröhlich, Berthold Stöger, Matthias Weil

**Affiliations:** a Institute of Applied Synthetic Chemistry, Vienna University of Technology, Getreidemarkt 9/163, A-1060 Vienna, Austria; b Institute for Chemical Technologies and Analytics, Division of Structural Chemistry, Vienna University of Technology, Getreidemarkt 9/164-SC, A-1060 Vienna, Austria

**Keywords:** Cyano­hydrin, cyclo­hexa-2,5-diene, crystal structure

## Abstract

The asymmetric unit of the title compound, C_14_H_22_N_2_O_2_Si_2_, contains one half of the mol­ecule, which is completed by inversion symmetry. The cyclo­hexa-2,5-diene ring is exactly planar and reflects the bond-length distribution of a pair of located double bonds [1.3224 (14) Å] and two pairs of single bonds [1.5121 (13) and 1.5073 (14) Å]. The tetra­hedral angle between the *sp* ^3^-C atom and the two neighbouring *sp* ^2^-C atoms in the cyclo­hexa-2,5-diene ring is enlarged by about 3°.

## Abstract 2

The mol­ecular structure of the title compound is centrosymmetric. The cyclo­hexa-2,5-diene moiety is exactly planar and has a bond-length distribution characteristic for one pair of double bonds and two pairs of single bonds.

## Chemical context   

Cyano­hydrins (Friedrich, 1983 ▶) are an important class of organic compounds. Silylated cyano­hydrins are versatile precursor compounds in organic chemistry because the nitrile functional group can be modified by a variety of reactions such as hydrolysis, reduction or addition of organometallic reagents. The mol­ecular and crystal structure of the title compound, a new silylated cyclo­hexa-2,5-diene with *trans* nitrile groups in the 1,4 positions, is reported herein.
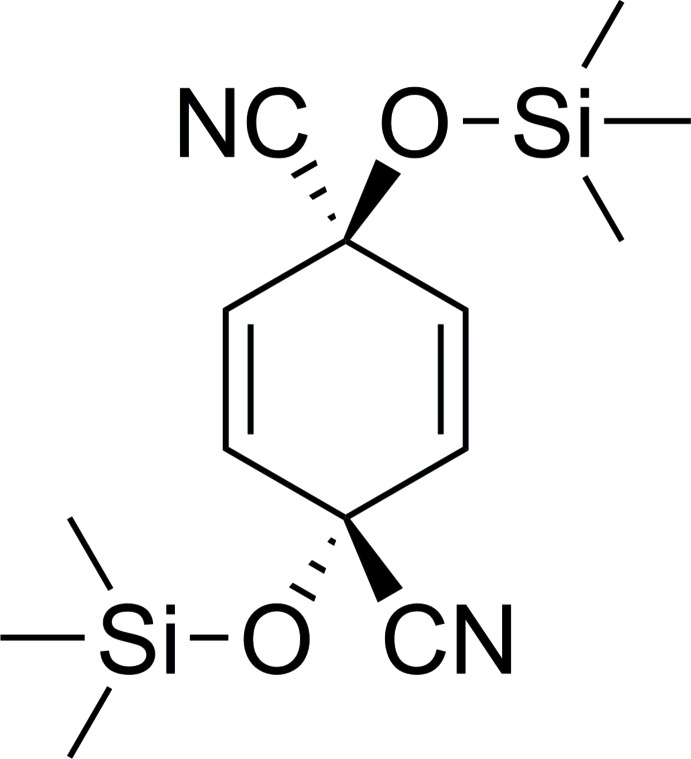


## Structural commentary   

**Figure 1:**
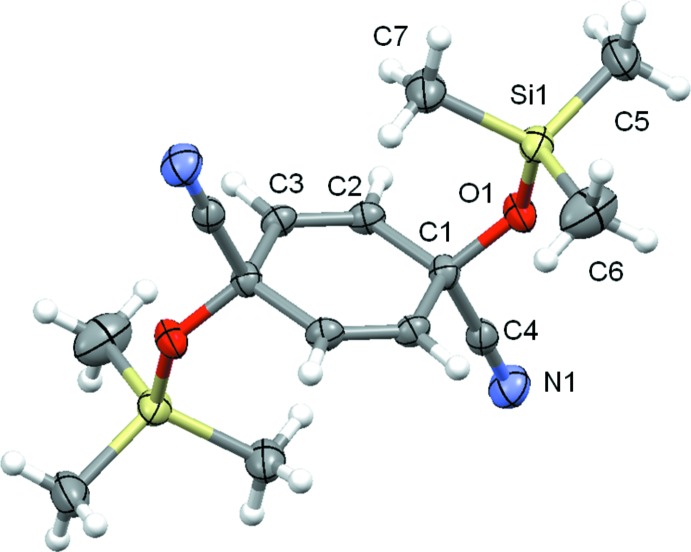
The mol­ecular structure of the title compound, showing the atom-labelling scheme and displacement ellipsoids drawn at the 80% probability level. Non-labelled atoms are generated by the symmetry code −*x* + 1, −*y* + 1, −*z*.

The mol­ecular structure of the title compound is centrosymmetric, leading to a *trans*-1,4-configuration of the oxy(tri­methyl­sil­yl) and carbo­nitrile groups (Fig. 1 ▶). The cyclo­hexa-2,5-diene ring is exactly planar, but its angles differ from that of an ideal hexa­gon. Whereas the angle between the *sp* ^3^-C atom (C1) and the neighbouring *sp* ^2^-C atoms (C2, C3) is reduced to 112.58 (8)°, the other intra-ring angles are enlarged to 123.94 (9)° (C1—C2—C3) and 123.48 (9)° (C1^i^—C3—C2) [symmetry code: (i) −*x* + 1, −*y* + 1, −*z*]. The tetra­hedral angles around C1 are likewise distorted due to the ring strain. The angles involving the O atom of the oxy(tri­methyl­sil­yl) group and the ring C atoms are enlarged to 110.79 (8)° and 113.26 (8)° while the angle involving the O atom and the C atom of the carbo­nitrile group is reduced to 104.95 (8)°. The backbone of the 1,1-substituents is nearly perpendicular to the cyclo­hexa-2,5-diene ring, with a dihedral angle of 86.05 (7).

## Supra­molecular features   

**Figure 2:**
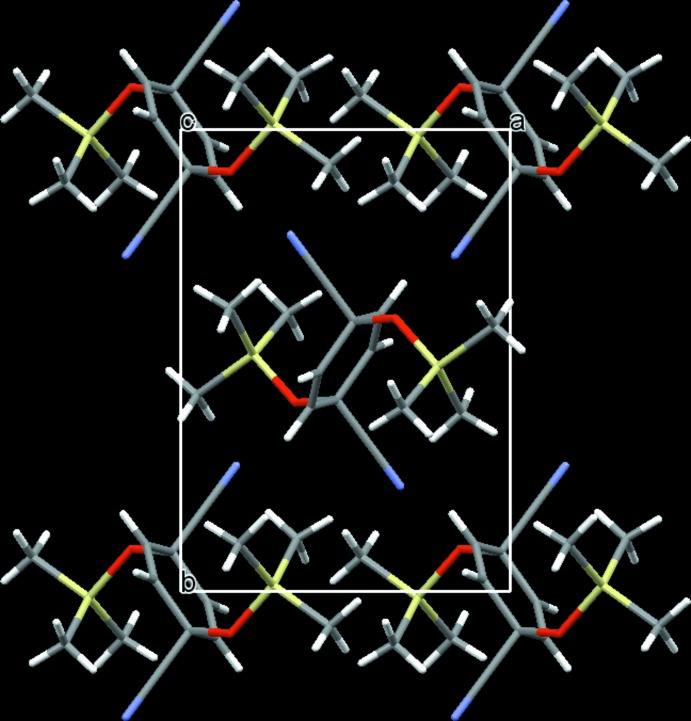
A view of the crystal packing of the title compound along [001]. Colour code: O red, C grey, N light-blue, Si off-white, H white.

Notable features in terms of non-classical hydrogen bonding inter­actions are not observed in the crystal structure of the title compound. As a result of the bulky tri­methyl­silyl groups, π–π stacking inter­actions between the rings are not possible. The packing of the mol­ecules (Fig. 2 ▶) seems to be dominated mainly by van der Waals forces.

## Database survey   

In the current Cambridge Structural Database (Version 5.35, last update February 2014; Allen, 2002 ▶) only one example of a cyclo­hexa-2,5-diene with *trans* nitrile groups in the 1,4 positions is listed, namely 3,5-bis­(4-(di­methyl­amino)­phen­yl)cyclo­hexa-2,5-diene-1,1,2,4,4-penta­carbo­nitrile (Jayamurugan *et al.*, 2011 ▶). The C—C bond lengths within the cyclo­hexa-2,5-diene are very similar to those of the title compound.

## Synthesis and crystallization   

1,4-Bis[(tri­methyl­sil­yl)­oxy]cyclo­hexa-2,5-diene-1,4-dicarbonitrile was synthesized by a modified protocol reported by Onaka *et al.* (1989 ▶). The required heterogeneous catalyst Fe-montmorillonite (K10-FeAA) was prepared according to Pai *et al.* (2000 ▶) and activated at 393 K and 5 mbar for 2 h prior to use.

**Figure 3:**
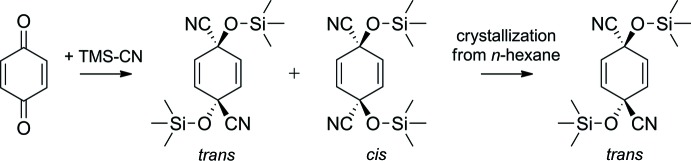
Reaction scheme to obtain the title compound.

1,4-Benzo­quinone (1.62 g, 15 mmol) was dissolved in 75 ml di­chloro­methane (0.2 M), purged with argon and cooled to 273 K. Tri­methyl­silyl cyanide (2.98 g, 30 mmol) and Fe-montmorillonite (0.75 g) were added sequentially and the mixture stirred for 1 h at 273 K under an argon atmosphere. The Fe-montmorillonite was filtered off (Por 4 glass filter) and the solvent was evaporated *in vacuo* to yield 4.23 g (13.8 mmol, 92%) of a *cis/trans* (3/1) isomeric mixture of 1,4-bis­[(tri­methyl­sil­yl)­oxy]cyclo­hexa-2,5-diene-1,4-dicarbo­nitrile (Fig. 3 ▶). Crystallization from *n*-hexane selectively yielded white crystals of the *trans*-isomer, which were suitable for single-crystal X-ray diffraction analysis. ^1^H NMR (CDCl_3_, 200 MHz): δ = 6.19 (*s*, 4H), 0.23 (s, 18H) p.p.m.; ^13^C NMR (CDCl_3_, 50 MHz): δ = 238.3 (*s*), 129.4 (*d*), 1.5 (*q*) p.p.m.

## Refinement   

Crystal data, data collection and structure refinement details are summarized in Table 1 ▶. The H atoms were included in calculated positions (C—H = 0.96 Å) and treated as riding atoms with *U*_iso_(H) = 1.2*U*_eq_(C).

## Supplementary Material

Crystal structure: contains datablock(s) general, I. DOI: 10.1107/S1600536814014251/su0009sup1.cif

Structure factors: contains datablock(s) I. DOI: 10.1107/S1600536814014251/su0009Isup2.hkl

Supporting information file. DOI: 10.1107/S1600536814014251/su0009Isup3.cml

CCDC reference: 1008752

Additional supporting information:  crystallographic information; 3D view; checkCIF report

## Figures and Tables

**Table 1:** Experimental details

Crystal data	
Chemical formula	C_14_H_22_N_2_O_2_Si_2_
*M*_r_	306.5
Crystal system, space group	Monoclinic, *P*2_1_/*n*
Temperature (K)	100
*a*, *b*, *c* (Å)	8.0770 (5), 11.2234 (6), 9.4377 (6)
β (°)	97.7087 (19)
*V* (Å^3^)	847.81 (9)
*Z*	2
Radiation type	Mo *K*α
μ (mm^−1^)	0.21
Crystal size (mm)	0.65 × 0.26 × 0.12

Data collection	
Diffractometer	Bruker Kappa APEXII CCD
Absorption correction	Multi-scan (*SADABS*; Bruker, 2013 ▶)
*T*_min_, *T*_max_	0.94, 0.98
No. of measured, independent and observed [*I* > 3σ(*I*)] reflections	15160, 2487, 2123
*R*_int_	0.024
(sin θ/λ)_max_ (Å^−1^)	0.705

Refinement	
*R*[*F* ^2^ > 3σ(*F* ^2^)], *wR*(*F* ^2^), *S*	0.030, 0.042, 2.38
No. of reflections	2487
No. of parameters	91
H-atom treatment	H-atom parameters constrained
Δρ_max_, Δρ_min_ (e Å^−3^)	0.38, −0.20

Computer programs: *APEX2* and *SAINT-Plus* (Bruker, 2013 ▶), *SUPERFLIP* (Palatinus & Chapuis, 2007 ▶), *JANA2006* (Petříček, *et al.*, 2014 ▶), *Mercury* (Macrae *et al.*, 2008 ▶) and *publCIF* (Westrip, 2010 ▶).

## supplementary crystallographic information

### Crystal data

**Table:**

C_14_H_22_N_2_O_2_Si_2_	*F*(000) = 328
*M**_r_* = 306.5	*D*_x_ = 1.200 Mg m^−^^3^
Monoclinic, *P*2_1_/*n*	Mo *K*α radiation, λ = 0.71073 Å
Hall symbol: -P 2yn	Cell parameters from 7267 reflections
*a* = 8.0770 (5) Å	θ = 2.8–29.9°
*b* = 11.2234 (6) Å	µ = 0.21 mm^−^^1^
*c* = 9.4377 (6) Å	*T* = 100 K
β = 97.7087 (19)°	Block, clear colourless
*V* = 847.81 (9) Å^3^	0.65 × 0.26 × 0.12 mm
*Z* = 2	

### Data collection

**Table:**

Bruker Kappa APEXII CCD diffractometer	2487 independent reflections
Radiation source: X-ray tube	2123 reflections with *I* > 3σ(*I*)
Graphite monochromator	*R*_int_ = 0.024
ω and φ–scans	θ_max_ = 30.1°, θ_min_ = 2.8°
Absorption correction: multi-scan (*SADABS*; Bruker, 2013)	*h* = −11→11
*T*_min_ = 0.94, *T*_max_ = 0.98	*k* = −15→15
15160 measured reflections	*l* = −13→13

### Refinement

**Table:**

Refinement on *F*	44 constraints
*R*[*F*^2^ > 2σ(*F*^2^)] = 0.030	H-atom parameters constrained
*wR*(*F*^2^) = 0.042	Weighting scheme based on measured s.u.'s *w* = 1/(σ^2^(*F*) + 0.0001*F*^2^)
*S* = 2.38	(Δ/σ)_max_ = 0.023
2487 reflections	Δρ_max_ = 0.38 e Å^−^^3^
91 parameters	Δρ_min_ = −0.20 e Å^−^^3^
0 restraints	

### Fractional atomic coordinates and isotropic or equivalent isotropic displacement parameters (Å^2^)

**Table:**

	*x*	*y*	*z*	*U*_iso_*/*U*_eq_	
Si1	0.21973 (4)	0.48658 (3)	0.25729 (3)	0.01515 (9)	
O1	0.34897 (9)	0.59176 (6)	0.20989 (8)	0.0151 (2)	
N1	0.66734 (12)	0.77227 (8)	0.18067 (10)	0.0203 (3)	
C1	0.47262 (12)	0.58514 (9)	0.11702 (10)	0.0118 (3)	
C2	0.39530 (12)	0.59953 (9)	−0.03704 (10)	0.0130 (3)	
C3	0.41937 (12)	0.52449 (9)	−0.14034 (11)	0.0125 (3)	
C4	0.58275 (13)	0.69117 (9)	0.15441 (10)	0.0136 (3)	
C5	0.04800 (15)	0.57505 (11)	0.31580 (13)	0.0244 (4)	
C6	0.32433 (16)	0.39773 (11)	0.40911 (13)	0.0303 (4)	
C7	0.14743 (14)	0.38628 (10)	0.10522 (12)	0.0208 (3)	
H1c2	0.324479	0.667221	−0.061301	0.0155*	
H1c3	0.365173	0.540694	−0.235238	0.015*	
H1c5	−0.036613	0.522349	0.341718	0.0293*	
H2c5	0.09082	0.622785	0.396915	0.0293*	
H3c5	0.000656	0.625942	0.239152	0.0293*	
H1c6	0.246714	0.341141	0.438738	0.0363*	
H2c6	0.417964	0.356223	0.379805	0.0363*	
H3c6	0.362472	0.449816	0.487404	0.0363*	
H1c7	0.049234	0.344384	0.124438	0.0249*	
H2c7	0.121741	0.432548	0.019545	0.0249*	
H3c7	0.233911	0.330007	0.093019	0.0249*	

### Atomic displacement parameters (Å^2^)

**Table:**

	*U*^11^	*U*^22^	*U*^33^	*U*^12^	*U*^13^	*U*^23^
Si1	0.01621 (16)	0.01632 (17)	0.01325 (15)	−0.00379 (11)	0.00316 (11)	−0.00069 (11)
O1	0.0166 (4)	0.0138 (4)	0.0161 (4)	−0.0014 (3)	0.0071 (3)	−0.0023 (3)
N1	0.0220 (5)	0.0178 (5)	0.0210 (5)	−0.0034 (4)	0.0026 (4)	−0.0036 (4)
C1	0.0132 (4)	0.0106 (5)	0.0118 (4)	−0.0006 (3)	0.0026 (3)	−0.0006 (3)
C2	0.0126 (4)	0.0112 (5)	0.0147 (5)	0.0009 (4)	0.0001 (4)	0.0016 (4)
C3	0.0126 (5)	0.0121 (5)	0.0122 (4)	0.0000 (4)	−0.0005 (4)	0.0017 (4)
C4	0.0151 (5)	0.0143 (5)	0.0115 (4)	0.0018 (4)	0.0021 (3)	−0.0004 (4)
C5	0.0226 (6)	0.0291 (7)	0.0236 (6)	−0.0046 (5)	0.0102 (5)	−0.0070 (5)
C6	0.0327 (7)	0.0314 (7)	0.0249 (6)	−0.0108 (5)	−0.0029 (5)	0.0105 (5)
C7	0.0215 (6)	0.0219 (6)	0.0198 (5)	−0.0064 (4)	0.0058 (4)	−0.0038 (4)

### Geometric parameters (Å, º)

**Table:**

Si1—C5	1.8495 (13)	C3—H1c3	0.96
Si1—C6	1.8537 (13)	C5—H1c5	0.96
Si1—C7	1.8555 (11)	C5—H2c5	0.96
O1—C1	1.4163 (13)	C5—H3c5	0.96
N1—C4	1.1451 (14)	C6—H1c6	0.96
C1—C2	1.5121 (13)	C6—H2c6	0.96
C1—C3^i^	1.5073 (14)	C6—H3c6	0.96
C1—C4	1.4993 (14)	C7—H1c7	0.96
C2—C3	1.3224 (14)	C7—H2c7	0.96
C2—H1c2	0.96	C7—H3c7	0.96
			
C5—Si1—C6	109.89 (6)	Si1—C5—H2c5	109.47
C5—Si1—C7	112.70 (5)	Si1—C5—H3c5	109.47
C6—Si1—C7	109.57 (5)	H1c5—C5—H2c5	109.47
O1—C1—C2	110.79 (8)	H1c5—C5—H3c5	109.47
O1—C1—C3^i^	113.26 (8)	H2c5—C5—H3c5	109.47
O1—C1—C4	104.95 (8)	Si1—C6—H1c6	109.47
C2—C1—C3^i^	112.58 (8)	Si1—C6—H2c6	109.47
C2—C1—C4	107.28 (8)	Si1—C6—H3c6	109.47
C3^i^—C1—C4	107.46 (8)	H1c6—C6—H2c6	109.47
C1—C2—C3	123.94 (9)	H1c6—C6—H3c6	109.47
C1—C2—H1c2	118.03	H2c6—C6—H3c6	109.47
C3—C2—H1c2	118.03	Si1—C7—H1c7	109.47
C1^i^—C3—C2	123.48 (9)	Si1—C7—H2c7	109.47
C1^i^—C3—H1c3	118.26	Si1—C7—H3c7	109.47
C2—C3—H1c3	118.26	H1c7—C7—H2c7	109.47
N1—C4—C1	178.87 (11)	H1c7—C7—H3c7	109.47
Si1—C5—H1c5	109.47	H2c7—C7—H3c7	109.47

Symmetry code: (i) −*x*+1, −*y*+1, −*z*.
